# John Wesley Shepherd

**Published:** 1855-07

**Authors:** S. M. Shepherd


Obituary.—My brother, John Wesley Shepherd, was born in Nelson county, Virginia, March, 1817. In the fall of 1834, being then 17 years old, he removed with my father and his family to the State of Ohio. At this time I had just entered upon the study of *dentistry* in my native State, Virginia, where I remained, completed my studies, and commenced practice in Petersburg. John in the meantime grew up, returned to Virginia, and entered my office for the purpose of obtaining a dental education—there being then no public institution for teaching dentistry. As a student he applied himself with diligence, and in a comparatively short time was well qualified to commence practice, for which he showed a strong inclination. He exhibited in his early practice an amount of skill and thoroughness in his operations which is rarely met with in those just entering professional life. In 1847, he married a lady in the county of Caroline, Virginia, whose family and family connections were numerous in the county, and whose influence might be turned to his account in the practice of his profession. This and other circumstances induced him to locate in Caroline. There he set himself vigorously to work to establish, not only himself, but a sound public sentiment in relation to the science of dentistry. To the latter it was needful that he should address himself with a bold hand; for the tyro and the impostor had traveled over the ground before him. The people, however, were intelligent, and able to comprehend sound doctrine. Here, although in comparative obscurity, he was a liberal patron of dental literature, and kept himself well posted in relation to the advancement of his art. He took great interest in every thing new that appeared in the dental publications from time to time; and although not a contributor, it may be gratifying to those who were, to know that he was a deeply interested beneficiary.

While in the State of Ohio, and a short time before he left for Virginia, he had an attack of *bilious fever,* from which he narrowly escaped with his life. There is ground for reasonable conjecture that the seeds of consumption were then planted, where they continued to take root until he finally fell a victim to that insidious disease. He was rarely free from uneasiness in his breast from the time of his recovery from the attack above referred to. During the last two or three years of his life the trouble in his breast became more afflictive—sometimes confining him to his bed for several days. Six months before his death he had so far lost his physical strength as to be unable to go out. From this time he rapidly failed, until, on the 7th of February last, he yielded up his spirit to God who gave it, in the 38th year of his age.

John was not only zealous in matters pertaining to his profession, but he was a christian. He embraced the religion of the Bible in the days of his childhood, and the Bible was his *chief* book. In his death, as well as his life, the consolations of the Gospel were abundantly manifested.

S. M. SHEPHERD.

